# Alpinetin Targets Mitophagy Pathways to Mitigate Parkinson's Disease Progression

**DOI:** 10.1002/cns.70676

**Published:** 2025-12-02

**Authors:** Zilu Shen, Xuesong Shan, Shenglan Zhang, Dan Huang, Haijun Hu, Yonglin Liang, Hong Zhu, Lieliang Zhang, Yayu Chen

**Affiliations:** ^1^ Departments of Anesthesiology, The Second Affiliated Hospital, Jiangxi Medical College Nanchang University Nanchang Jiangxi Province China; ^2^ The Second Clinical Medical College of Nanchang University Nanchang Jiangxi Province China; ^3^ Jiangxi Province Key Laboratory of Anesthesiology Nanchang Jiangxi Province China; ^4^ Department of Neurosurgery, The Second Affiliated Hospital, Jiangxi Medical College Nanchang University Nanchang China

**Keywords:** Alpinetin, mitophagy, network pharmacology, parkinson's disease

## Abstract

**Aims:**

Parkinson's disease (PD) is a complex neurodegenerative disorder lacking disease‐modifying therapies. This study aimed to systematically investigate the therapeutic potential and underlying mechanisms of Alpinetin in PD.

**Methods:**

An integrated approach combining network pharmacology and molecular docking was employed to predict the core targets and pathways of Alpinetin in PD. These computational predictions were subsequently validated through in vivo animal experiments.

**Results:**

Network pharmacology analysis predicted that Alpinetin exerts its effects by modulating mitophagy and dopaminergic synaptic pathways. Molecular docking revealed strong binding affinities between Alpinetin and key targets, including HIF1A, SQSTM1, and SRC. Guided by these findings, animal experiments confirmed the neuroprotective effects of Alpinetin, aligning with the predicted mechanisms.

**Conclusion:**

Our findings demonstrate that Alpinetin has significant therapeutic potential for PD, likely mediated through the regulation of mitophagy and dopaminergic synapses. This study elucidates the molecular targets and mechanisms of Alpinetin, providing a solid foundation for its further investigation as an anti‐PD agent.

## Graphical abstract

Combining network pharmacology, molecular docking, and in vivo studies, we demonstrate that Alpinetin exerts neuroprotective effects in PD by ameliorating mitophagy dysfunction and preserving dopaminergic synaptic integrity.

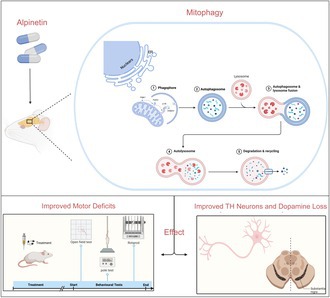

**Abbreviations**
BP: biological processes
CC: cellular components
CTD: comparative toxicogenomic database
DA: dopamine
DC: dendritic cell
GO: gene ontology
HPLC: high‐performance liquid chromatography
HRP: horseradish peroxidase
KEGG: Kyoto Encyclopedia of Genes and Genomes
LRRK2: leucine‐rich repeat kinase 2
MF: molecular functions
NP: network pharmacology
OMIM: the Online Mendelian Inheritance in Man
PBS: phosphate‐buffered saline
PD: Parkinson's disease
PFA: paraformaldehyde
PPARγ: peroxisome proliferator‐activated receptor gamma
PPI: protein–protein interaction
ROS: reactive oxygen species
SNPC: substantia nigra pars compacta
TCM: traditional Chinese medicine
TH: tyrosine hydroxylase
TTD: therapeutic target database

## 1 Introduction

Parkinson's disease (PD) is a long‐term, progressively worsening neurodegenerative disorder marked mainly by the loss of dopaminergic neurons in the substantia nigra [1]. This neuronal decline results in a substantial decrease in dopamine (DA), a critical neurotransmitter for motor coordination and movement control. As the disease progresses, patients commonly exhibit hallmark symptoms, including bradykinesia, rigidity, tremor, and gait and postural disturbances [2]. In response to these motor symptoms, levodopa is typically used for treatment in the early stages, and it can also improve some non‐motor symptoms, such as depression and anxiety, which may exhibit similar fluctuating patterns [3]. However, the long‐term use of levodopa is associated with diminishing efficacy and the emergence of side effects, including motor fluctuations and dyskinesias [4]. Furthermore, these treatments do not address the underlying neurodegenerative processes, underscoring a critical gap in effective therapeutic strategies for PD. In light of these limitations, there is increasing interest in alternative therapies, particularly traditional Chinese medicine (TCM). Recent studies have demonstrated that TCM may modulate various pathophysiological aspects of PD, such as neuroinflammation, reactive oxygen species metabolism and apoptosis [5, 6], etc., thereby offering potential neuroprotective effects [7]. Given these findings and the broader context of PD pathology, exploring additional therapeutic pathways through TCM may provide new insights into its potential efficacy in managing the disease.

Alpinetin is a natural flavonoid compound primarily found in plants of the ginger family [8], traditionally used in Chinese medicine to treat various digestive disorders, including epigastric pain, hiccups, nausea, vomiting, and anorexia. Previous studies have demonstrated that Alpinetin exhibits a broad spectrum of pharmacological activities, such as anti‐cancer, anti‐inflammatory, cardiovascular protective, antibacterial, and antiviral effects [8]. Furthermore, Alpinetin has shown potential in the treatment of nervous system disorders [9]. Studies have demonstrated that Alpinetin inhibits LPS‐induced inflammatory mediator production by activating PPAR‐γ and suppressing TLR4 expression [10]. Additionally, Alpinetin reduces neuroinflammation and neuronal apoptosis through modulation of the JAK2/STAT3 pathway [11]. Neuroinflammation contributes to dopamine neuron damage and plays a key role in the pathogenesis of PD [12]. In aging‐related cognitive impairment, Alpinetin mitigates mitochondrial inflammation via the Drp1/HK1/NLRP3 pathway [13]. Notably, mitochondrial dysfunction synergistically interacts with synaptic and lysosomal damage in PD progression [14]. These findings highlight Alpinetin's therapeutic potential for PD, but the precise molecular mechanisms underlying these effects remain to be fully elucidated.

Mitochondrial dysfunction has increasingly been identified as a key contributor to PD pathology [15]. As essential organelles in cellular energy production, mitochondria, when damaged, can cause reduced ATP levels and elevated oxidative stress, ultimately contributing to the degeneration of dopaminergic neurons [16, 17]. Thus, the maintenance of mitochondrial health is essential for neuronal survival and function. An important cellular process that supports mitochondrial integrity is mitophagy, the selective degradation of damaged or dysfunctional mitochondria [18]. Through this process, the accumulation of damaged mitochondria can be effectively prevented, thereby alleviating oxidative damage and maintaining cellular homeostasis [19]. In the study of PD, there is evidence that the decline of mitophagy is closely related to disease progression, and the accumulation of damaged mitochondria may lead to neuronal dysfunction and death [20]. Studies in animal models have demonstrated that impaired mitophagy plays a role in PD development, leading to the identification of various mechanisms and pharmacological agents targeting the mitophagy pathway as potential therapeutic strategies [17]. Therefore, enhancing mitophagy function may represent an emerging therapeutic strategy for PD.

To elucidate the potential therapeutic mechanisms of PD, we utilized network pharmacology. In 2007, British scholar Andrew L. Hopkins first introduced the concept of network pharmacology (NP) [21]. Network pharmacology is an interdisciplinary field that integrates pharmacology, systems biology, information network theory, and computer science, utilizing computational simulations and diverse databases to identify molecular drug targets and disease‐related targets [22]. Through high‐throughput screening, network visualization, and network analysis, NP uncovers the intricate interactions between drugs, targets, and diseases, enabling the prediction and analysis of drug mechanisms of action. These predictions are further validated experimentally when necessary [23]. NP is particularly effective in exploring connections between the multiple components of traditional Chinese medicine and disease‐related targets, making it widely applicable in Chinese medicine research.

Molecular docking is a technique in computational biology used to predict how small molecules, like drugs, interact with target biomolecules, such as proteins and DNA, by analyzing their binding modes and affinities. By evaluating the energies and interactions of various binding conformations, molecular docking aids researchers in identifying potential drug candidates and provides critical insights for drug design. This approach is integral to drug discovery and development, significantly expediting the overall drug development process [24].

**FIGURE 1:**
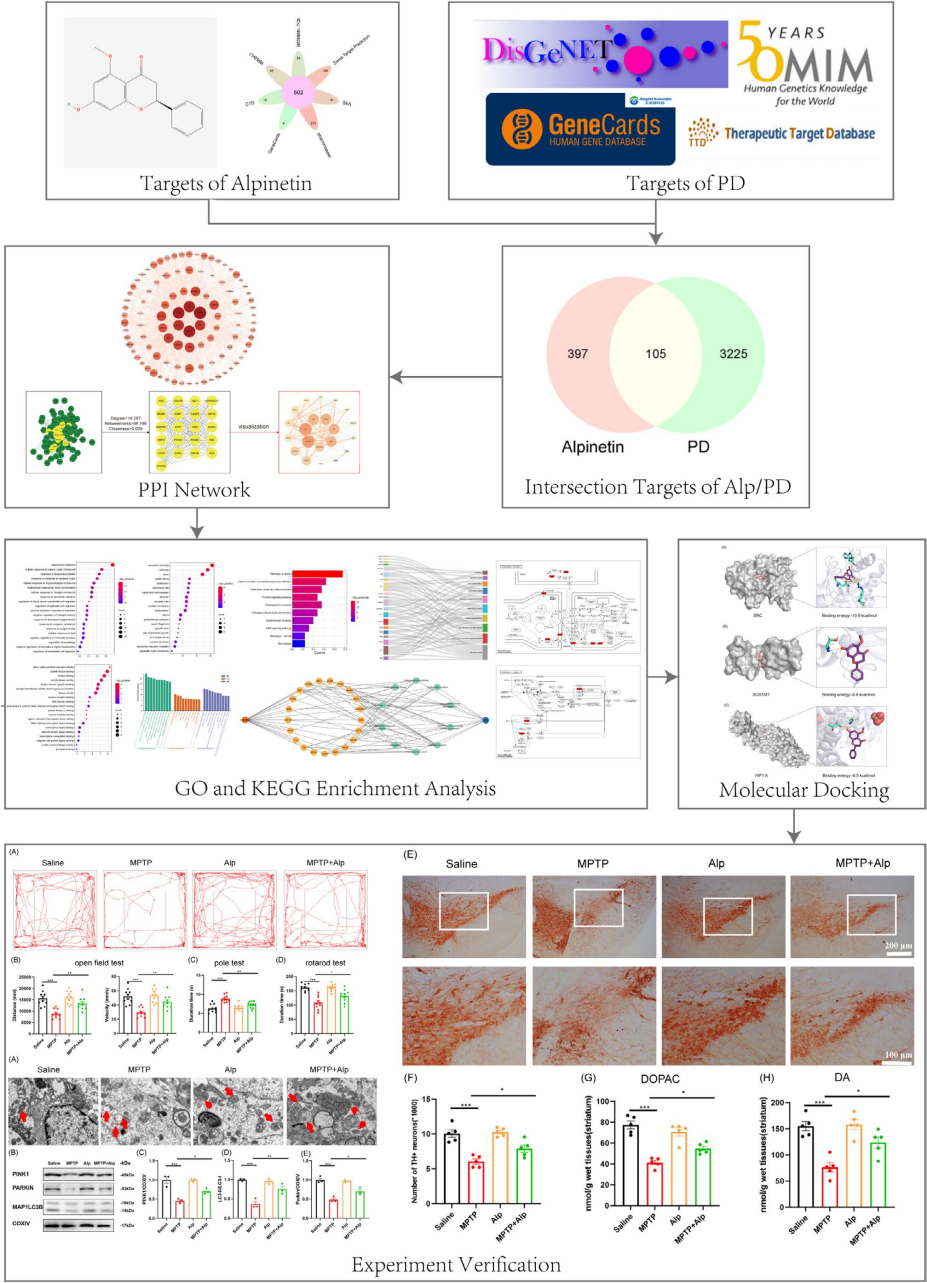
Flow chart of action mechanism of Alpinetin against PD.

In this study, network pharmacology and molecular docking were utilized to identify the main components and prospective targets of Alpinetin for PD treatment. The effects of Alpinetin were further validated using the MPTP‐induced mouse model, providing experimental data for future research. The study workflow is illustrated in Figure 1.

## 2 Material and Method

### 2.1 Collecting the Genes of PD

We retrieved PD‐associated genes from multiple databases, including the Online Mendelian Inheritance in Man (OMIM) database [25], the GeneCards database [26], the DisGeNet database [27], and the Therapeutic Target Database (TTD) [28]. The PD genes were identified by searching for “Parkinson Disease” in each database.

### 2.2 Target Prediction for Alpinetin and Identification of Intersection Targets for Anti‐PD Action

We retrieved chemical information for Alpinetin from the PubChem database and identified potential targets using seven databases: PharmMapper [29], Swiss Target Prediction [30], GeneCards [26], ChEMBL [31], SEA [32], BATMAN‐TCM [33], and the Comparative Toxicogenomic Database (CTD) [34]. By searching for “Alpinetin” or entering its molecular formula or SMILES code, we obtained drug targets from each database. To identify overlapping targets between PD and Alpinetin, we used the Microbioinformatics online platform [35] to construct a Venn diagram of PD‐associated genes and Alpinetin‐related targets.

### 2.3 Core Target Selection Using PPI Network Interaction Analysis

We imported the intersecting targets of Alpinetin and PD into the STRING database [26] to construct a protein–protein interaction (PPI) network, selecting “ *Homo sapiens* ” as the species and setting the confidence score to “medium confidence > 0.4.” The PPI network was exported and visualized using Cytoscape 3.7 software. Core targets were identified using the CentiScape plugin, a robust tool for target filtering. Three commonly used algorithms—degree, betweenness, and closeness—were applied to rank the targets, and the top‐ranked targets from each algorithm were selected as core candidate targets.

### 2.4 GO and KEGG Enrichment Analysis

We performed Gene Ontology (GO) functional and Kyoto Encyclopedia of Genes and Genomes (KEGG) pathway enrichment analyses on the key targets using the Metascape database [36]. The enrichment criteria were set at *p* < 0.01, with a minimum of three enriched genes per category. The top 20 significant biological processes (BP), cellular components (CC), molecular functions (MF), and the top 10 KEGG pathway enrichment results were visualized using the Microbioinformatics online platform [35]. Additionally, we constructed a drug‐target–KEGG pathway–disease interaction network using Cytoscape 3.7 software.

### 2.5 Molecular Docking

Key targets of Alpinetin for PD treatment were identified based on the PPI network and enrichment analysis and subsequently selected for molecular docking. The active compound structures of Alpinetin were retrieved from the TCMSP database in mol2 format, then prepared and converted to pdbqt format using AutoDockTools 1.5.6. The 3D crystal structures of target proteins were obtained from the PDB database (https://www.rcsb.org/) with the following PDB IDs: HIF1A (8he3), SQSTM1 (5yp7), and SRC (1us0). Using PyMOL, water molecules and organic matter were removed from the target proteins, followed by hydrogenation, charge assignment, and atom type addition in AutoDockTools 1.5.6. The files were saved in pdbqt format. Molecular docking was performed with AutoDock Vina, and the results were visualized with PyMOL.

### 2.6 Experimental Validation

#### 2.6.1 Reagents

Reagents purchase information was as follows: 1‐methyl‐4‐phenyl‐1,2,3,6‐tetrahydropyridine (MPTP, Sigma‐Aldrich, USA, M0896), Probenecid (Selleck Chemicals, S4022), DMSO (Selleck Chemicals, B14001), Alpinetin (Selleck Chemicals, S3875), Mitochondrial Protein Extraction Kit (Beyotime Biotechnology, China, C3606), Anti‐Tyrosine Hydroxylase Antibody (TH) (1:1000, Millipore, AB152), Horseradish Peroxidase (HRP, Invitrogen, 61‐6520), Diaminobenzidine (DAB, Thermo Scientific, 34002), Phenyl methane sulfonyl fluoride (PMSF, Thermo Scientific, 36978).

#### 2.6.2 Model Establishment and Animal Management

Male mice were selected for MPTP model establishment in this study due to their heightened sensitivity to dopaminergic neuron damage and greater striatal dopamine depletion compared to female mice, characteristics that optimize model validity. Furthermore, the male model minimizes confounding variables associated with cyclic estrogen fluctuations in females, thereby enhancing experimental stability and result reproducibility [37]. MPTP is a neurotoxin frequently used to create animal models of PD. In this study, healthy male adult mice of comparable age, and weight were chosen and randomly assigned to one of four groups: Control group, MPTP‐induced model group, Alpinetin group (drug‐only group), and MPTP + Alpinetin group (treatment group). To induce a chronic PD model, the mice received 11 injections of MPTP and probenecid, dissolved in DMSO, over a period of 5.5 weeks, with injections administered every 3.5 days [38]. Each 20 mg/kg MPTP injection was preceded by a 250 mg/kg intraperitoneal injection of probenecid, administered 30 min earlier. The control group received equivalent subcutaneous saline injections and intraperitoneal DMSO injections every 30 min. Behavioral assessments using the rotarod test were conducted after the final MPTP/probenecid injection. Only mice displaying behavioral impairments were selected for further experiments.

To assess the protective effects and mechanisms of Alpinetin in a chronic PD model, mice were randomly divided into four groups: (1) control group (saline), (2) MPTP/probenecid model group, (3) Alpinetin group (Alpinetin 25 mg/kg dissolved in DMSO), and (4) MPTP/probenecid + Alpinetin group (Alpinetin 25 mg/kg dissolved in DMSO). Mice in the Alpinetin‐treated groups were pre‐treated with Alpinetin every other day for 1 week before co‐treatment with MPTP, which continued with the same frequency for an additional week.

#### 2.6.3 Behavioral Testing

##### 2.6.3.1 Open Field Test

A 40 cm × 40 cm × 40 cm open‐top box was used to allow mice to explore freely for 5 min. During the test, their movement paths, distance traveled, and time spent in various areas were recorded to evaluate levels of locomotor activity [39].

##### 2.6.3.2 Pole Test

Mice were trained to climb a pole for three consecutive days prior to testing, with three training sessions each lasting 1 h. A 50 cm high pole, topped with a 25 mm diameter ball, was used, and a cushion was placed at the bottom to prevent injury. On the third day, mice were placed head‐up at the top of the pole, and the time taken to descend was recorded to evaluate their descending speed [39].

##### 2.6.3.3 Rotarod Test

Mice underwent training on a slowly rotating rod for three consecutive days before the test. During testing, the rod's speed was progressively increased from 4 rpm to 40 rpm over a duration of 3 min. The time each mouse stayed on the rod without falling was recorded as a measure of muscle coordination and balance [40].

#### 2.6.4 Immunohistochemistry (IHC)

Mice were anesthetized using sodium pentobarbital and transcardially perfused with phosphate‐buffered saline (PBS) to clear blood, followed by 4% paraformaldehyde (PFA) in PBS to fix tissues. The entire brain was then excised, dehydrated, and sectioned. Midbrain sections of approximately 30 μm thickness were prepared using a microtome, mounted onto gelatin‐coated slides to ensure adhesion, and stored at −20°C until further use. Sections were initially washed in PBS and permeabilized with 0.3% Triton X‐100 in PBS for 10 min to enhance antibody penetration. To minimize non‐specific binding, sections were incubated in 5% goat serum prepared in PBS for 1 h at room temperature. Following this blocking step, sections were incubated overnight at 4°C with a primary antibody specific to tyrosine hydroxylase (TH) (rabbit anti‐TH, 1:1000). The next day, sections were rinsed in PBS to remove unbound primary antibody and incubated with a goat anti‐rabbit IgG secondary antibody conjugated to horseradish peroxidase (HRP) for 1 h at room temperature. After a final PBS wash, sections were incubated with a chromogenic substrate, diaminobenzidine (DAB), which reacts with the HRP conjugate to produce a brown precipitate marking TH‐positive neurons. Sections were counterstained with hematoxylin to visualize nuclei, dehydrated, and mounted. Stained sections were examined under a light microscope, and images of TH‐positive neurons in the substantia nigra were captured.

#### 2.6.5 High‐Performance Liquid Chromatography (HPLC)

The striatum was dissected and immediately placed in pre‐chilled saline to prevent tissue degradation. The tissue was homogenized at 4°C using a buffer containing mannitol, sucrose, EDTA, and HEPES. Organic solvents (acetonitrile or methanol) were added to precipitate proteins, followed by centrifugation at 12,000 rpm for 15 min to collect the supernatant. The supernatant was filtered, typically through a 0.2‐μm or smaller filter, to remove any particles that could block the chromatography column. An HPLC system equipped with a C18 reverse‐phase column and a gradient mobile phase (water/acetonitrile with 0.1% phosphoric acid) was used. Dopamine and its metabolites were detected using an electrochemical detector with a potential of 800 mV. The sample injection volume was 20 μL, and the flow rate was 1 mL/min. The neurotransmitter content in the sample was calculated using the fundamental standard curve and the external standard method.

#### 2.6.6 Transmission Electron Microscopy

Under appropriate anesthesia, mice were euthanized, and midbrain tissue was swiftly excised and immersed in 2.5% glutaraldehyde fixative for 24 h. Following fixation, tissues were dehydrated through a graded ethanol series, cleared with propylene oxide, and embedded in resin. Ultra‐thin sections (60–90 nm) were then cut using an ultramicrotome and stained with uranyl acetate and lead citrate. Imaging was performed using a transmission electron microscope at an accelerating voltage of 80 kV.

#### 2.6.7 Mitochondrial Protein Extraction From Tissue

Following the manufacturer's protocol, all reagents from the Mitochondrial Protein Extraction Kit were thawed at room temperature, promptly placed on ice, and thoroughly mixed. For initial preparation, 1.5 mL of PMSF solvent was added to PMSF crystals to prepare a 100 mM stock solution, stored at −20°C. To prevent protease activity, PMSF was added to Mitochondrial Isolation Buffer A and Mitochondrial Lysis Buffer just before use, reaching a final concentration of 1 mM Fresh midbrain tissue from mice was weighed in a 1.5 mL centrifuge tube, briefly rinsed in PBS to remove impurities, and blotted to eliminate excess PBS. The tissue was placed into a pre‐chilled container on ice and finely minced with scissors. Ten volumes of pre‐cooled Mitochondrial Isolation Buffer A (containing 1 mM PMSF) were added, and the sample was homogenized on ice with approximately 10 strokes to ensure even dispersion. The homogenate was centrifuged at 600 × g for 5 min at 4°C to remove cellular debris, with the supernatant transferred to a new tube and centrifuged at 11,000×*g* for 10 min at 4°C to pellet the mitochondria. After discarding the supernatant, the mitochondrial pellet was retained. For the cytoplasmic protein fraction, the supernatant was centrifuged again at 12,000 × g for 10 min at 4°C, and the resulting supernatant was collected as the cytoplasmic protein sample. Protein concentration was measured using the Bradford assay, with standards prepared in PBS containing the same proportion of Mitochondrial Isolation Buffer.

#### 2.6.8 Western Blot

The isolated mitochondrial proteins were combined with SDS sample buffer and heated at 95°C for 5–10 min to denature the proteins. The prepared samples, along with a protein marker, were loaded into an SDS‐PAGE gel for electrophoresis until the proteins were completely separated. Proteins were then transferred from the gel to a nitrocellulose membrane, which was blocked with 5% BSA for 1 h. The membrane was incubated with specific primary antibodies overnight at 4°C. Following three washes with TBST, the membrane was incubated with appropriate secondary antibodies for 1 h at room temperature, then washed and detected using chemiluminescence. Protein sizes were determined by comparing band positions to molecular weight markers, and band intensity was used for quantitative analysis, allowing comparison of target protein abundance between samples.

#### 2.6.9 Statistical Analysis

Data are expressed as mean ± standard deviation (SD). Statistical analyses were conducted using GraphPad Prism 8.0 software. For comparisons between two groups, an unpaired *t*‐test was used, whereas one‐way analysis of variance (ANOVA) with Tukey's post hoc test was applied for comparisons among multiple groups. A *p*‐value of < 0.05 was considered to indicate statistical significance. All statistical results are explicitly reported, including test statistics and degrees of freedom, to enhance reproducibility.

## 3 Results

### 3.1 Collection of PD Genes

To comprehensively investigate the risk genes and elucidate the pathogenesis of PD, we collected gene targets from multiple databases: 474 from OMIM, 10,342 from GeneCards, 2078 from DisGeNet, and 57 from TTD. To enhance relevance, we filtered the 10,342 targets from GeneCards with a relevance score threshold of > 1.0126, yielding 2613 high‐priority targets. Similarly, we refined the 2078 DisGeNet targets by applying a minimum score of > 0.02, resulting in 1041 relevant targets. After removing duplicates across all datasets, we identified a total of 3330 unique PD‐related targets.

### 3.2 Prediction of Alpinetin Targets and Acquisition of Intersection Targets

**FIGURE 2:**
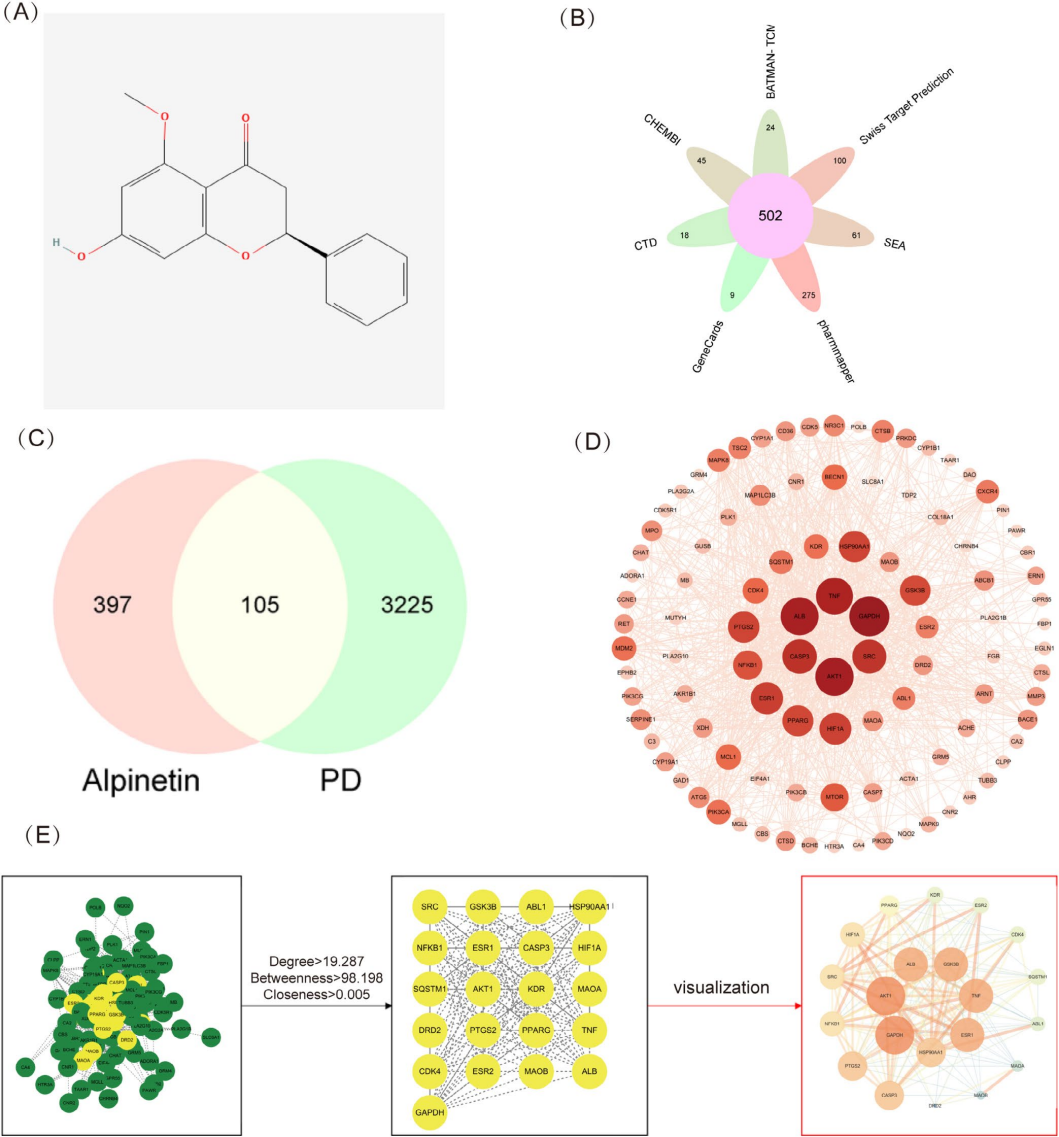
Network Pharmacology Analysis and Target Prediction of Alpinetin in PD. (A) Two‐dimensional structure of Alpinetin. (B) The number of Alpinetin candidate targets obtained from seven databases. (C) Venn diagram of common targets between Alpinetin and PD. (D) PPI network of common targets; the color and area of the target are proportional to the Degree value. (E) Core target identification process from the common targets.

As a natural flavonoid with notable anti‐inflammatory, antioxidant, and neuroprotective properties, Alpinetin has demonstrated therapeutic potential in various neurological conditions. However, its specific effects on PD remain largely unexplored. To address this gap and establish an experimental foundation for subsequent clinical treatment, we retrieved the two‐dimensional structure of Alpinetin from the PubChem database (Figure 2A) and identified Alpinetin‐related targets across multiple databases, including Pharm Mapper, Swiss Target Prediction, SEA, CHEMBI, BATMAN, CTD, and GeneCards. After removing duplicates, we obtained a final set of 502 unique drug targets (Figure 2B). To further explore potential therapeutic connections with PD, we constructed a Venn diagram using a bioinformatics platform, revealing 105 targets common to both Alpinetin and PD (Figure 2C).

### 3.3 Core Target Screening Using PPI Network Interaction Analysis

The 105 intersecting targets of Alpinetin and PD were imported into the STRING database to create a PPI network, with “ *Homo sapiens* ” specified as the organism and a confidence level set to “medium confidence > 0.4.” After removing isolated nodes unconnected to other targets, the PPI network contained 101 nodes and 974 edges (Figure 2D). Cytoscape 3.7 software was used to visualize the network, and core targets were identified using the CentiScape plugin, which assesses degree, closeness, and betweenness values. This process produced a refined PPI network with 21 nodes and 166 edges, visualized again in Cytoscape 3.7 (Figure 2E). Among the 21 core targets identified were NFKB1, KDR, and ALB, etc. (Table 1).

**TABLE 1:** Table of candidate target parameters.

Name	Betweenness	Closeness	Degree
NFKB1	99.90013	0.00625	42
KDR	151.4819	0.005747	31
ALB	750.7	0.007194	62
CASP3	300.0301	0.006849	54
GSK3B	329.6615	0.006452	45
HSP90AA1	281.9579	0.006494	47
PTGS2	519.7388	0.006494	47
ABL1	160.4764	0.00565	27
SRC	932.8894	0.006623	53
GAPDH	904.1321	0.007519	67
TNF	763.1666	0.007092	60
ESR1	376.2884	0.006623	49
HIF1A	332.2055	0.006494	48
AKT1	657.6327	0.007299	63
CDK4	181.8991	0.005848	34
SQSTM1	226.1051	0.005714	29
PPARG	324.0575	0.00641	46
DRD2	236.2945	0.005236	20
MAOA	149.7243	0.005376	22
MAOB	126.5479	0.005319	20
ESR2	133.6745	0.005618	27

### 3.4 GO and KEGG Enrichment Analysis

**FIGURE 3:**
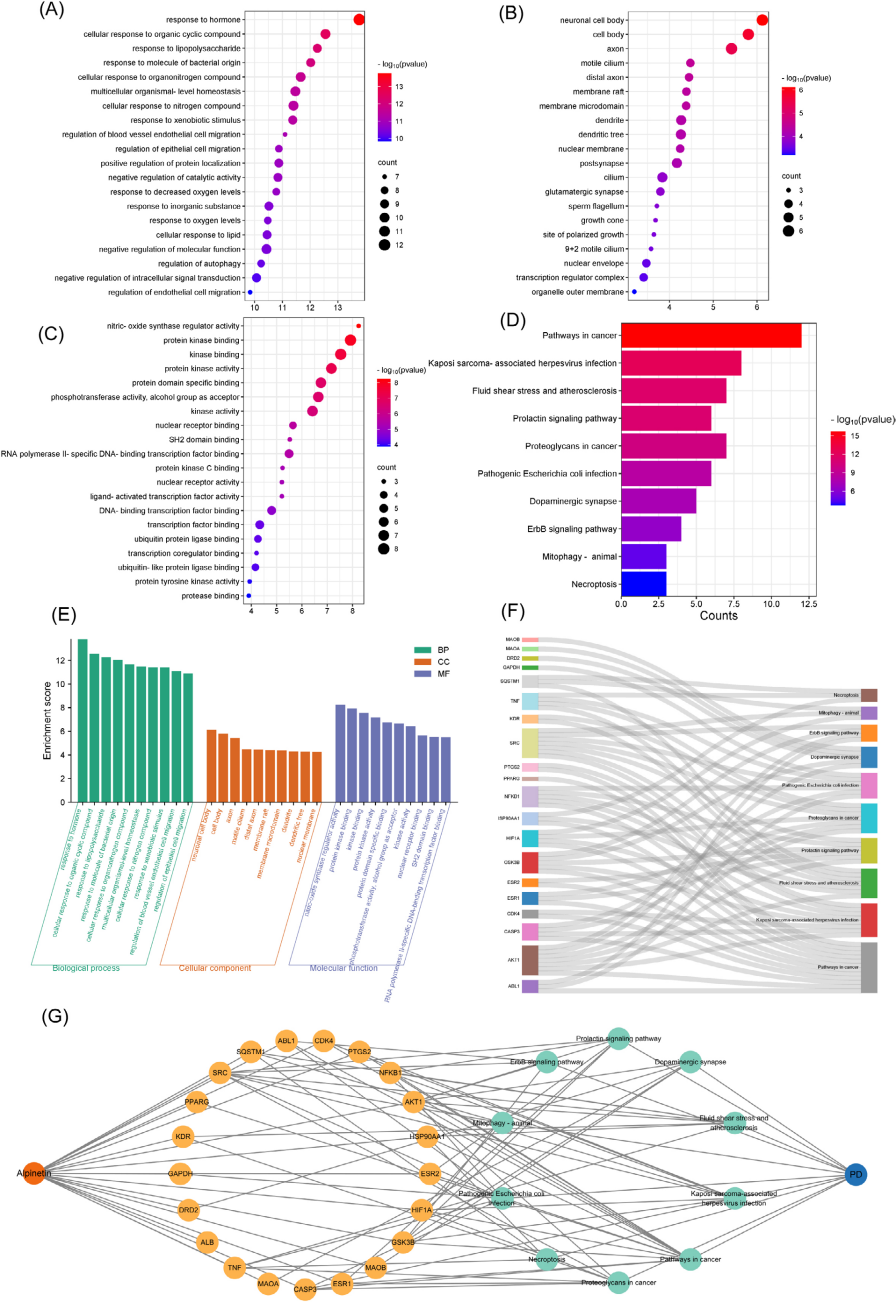
GO function and KEGG pathway enrichment analysis of key targets. (A) The top 20 GO items in BP. (B) The 20 GO items in CC. (C) The top 20 GO items in MF. (D) The top 10 KEGG pathways. BP, biological processes; CC, cellular components; GO, Gene Ontology; KEGG, Kyoto Encyclopedia of Genes and Genomes; MF, molecular functions. (E) Top 10 BP terms, CC terms, and MF terms of GO enrichment analysis are shown as green, orange, and purple bars, respectively. (F) Sankey diagram of target‐pathway‐disease associations in Alpinetin‐PD analysis. (G) The Sankey diagram of the KEGG pathway analysis of the therapeutic targets of Alpinetin in PD treatment. The left rectangle nodes of the Sankey diagram represent the therapeutic targets, the right rectangle nodes of the Sankey diagram represent the KEGG pathways, and the lines represent the ownership of targets and pathways.

To investigate the physiological roles of these genes, we conducted enrichment analysis on the core targets. The GO framework facilitates the identification of functional commonalities by detecting significantly enriched biological functions. Concurrently, KEGG enrichment analysis identified key metabolic and signaling pathways associated with these genes, elucidating their potential roles within complex regulatory networks. Core targets were submitted to the Metascape platform for GO and KEGG pathway enrichment analyses. Enrichment thresholds were set at *p* < 0.01 and a minimum gene count of ≥ 3, resulting in 626 GO terms and 92 KEGG pathways. From the GO analysis, the 20 terms with the lowest *p*‐values were selected across the categories of BP (Figure 3A), CC (Figure 3B), and MF (Figure 3C). Combined with enrichment scores and gene counts, the top 10 entries in each category are presented as bars (Figure 3E). The main BP entries are regulation of autophagy, response to decreased oxygen levels and cellular response to lipid. The CC entries mainly include neuronal cell body and dendrite. The MF entries are involved in protein kinase binding, transcription factor binding and nuclear receptor binding.

**FIGURE 4:**
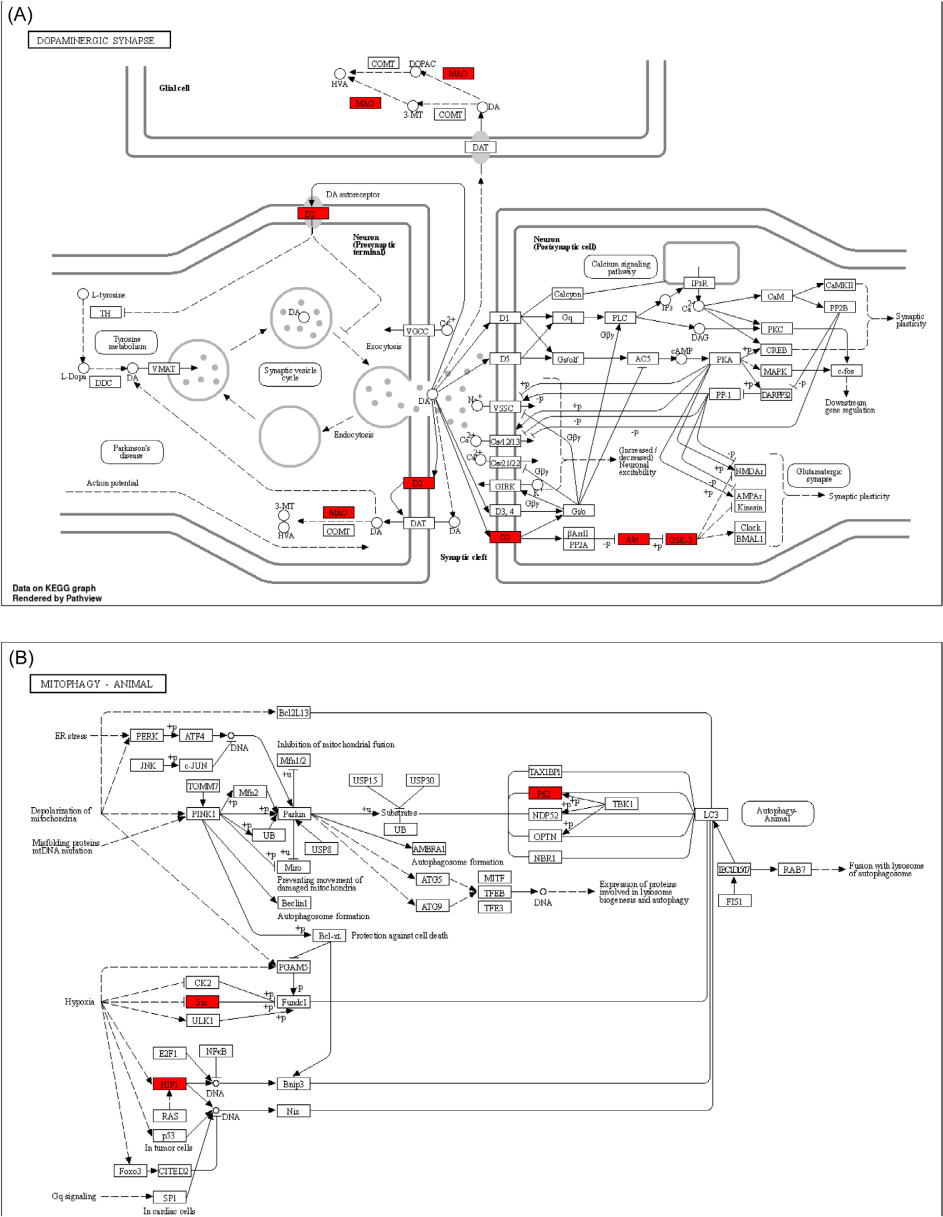
KEGG maps of the key target distribution. (A) The dopaminergic synapse pathway. (B) The mitophagy‐animal pathway. The white represents other targets of the pathway, and red represents the key target of Alpinetin against PD. KEGG, Kyoto Encyclopedia of Genes and Genomes; PD, Parkinson's disease.

The KEGG pathway enrichment highlighted several significant clusters, including the prolactin signaling pathway, proteoglycans in cancer, pathogenic *Escherichia coli* infection, dopaminergic synapse, ERB signaling pathway, mitophagy, and necrosis. Pathways primarily related to PD development included the dopaminergic synapse and mitophagy (Figure 3D,F). The Alpinetin‐target‐KEGG‐PD interaction network was visualized using Cytoscape 3.7 software (Figure 3G). Additionally, using a bioinformatics platform, the distribution of key targets in the dopaminergic synapse and mitophagy pathways was mapped on the animal KEGG pathways, with the main targets highlighted in red (Figure 4).

### 3.5 Molecular Docking Verification

**FIGURE 5:**
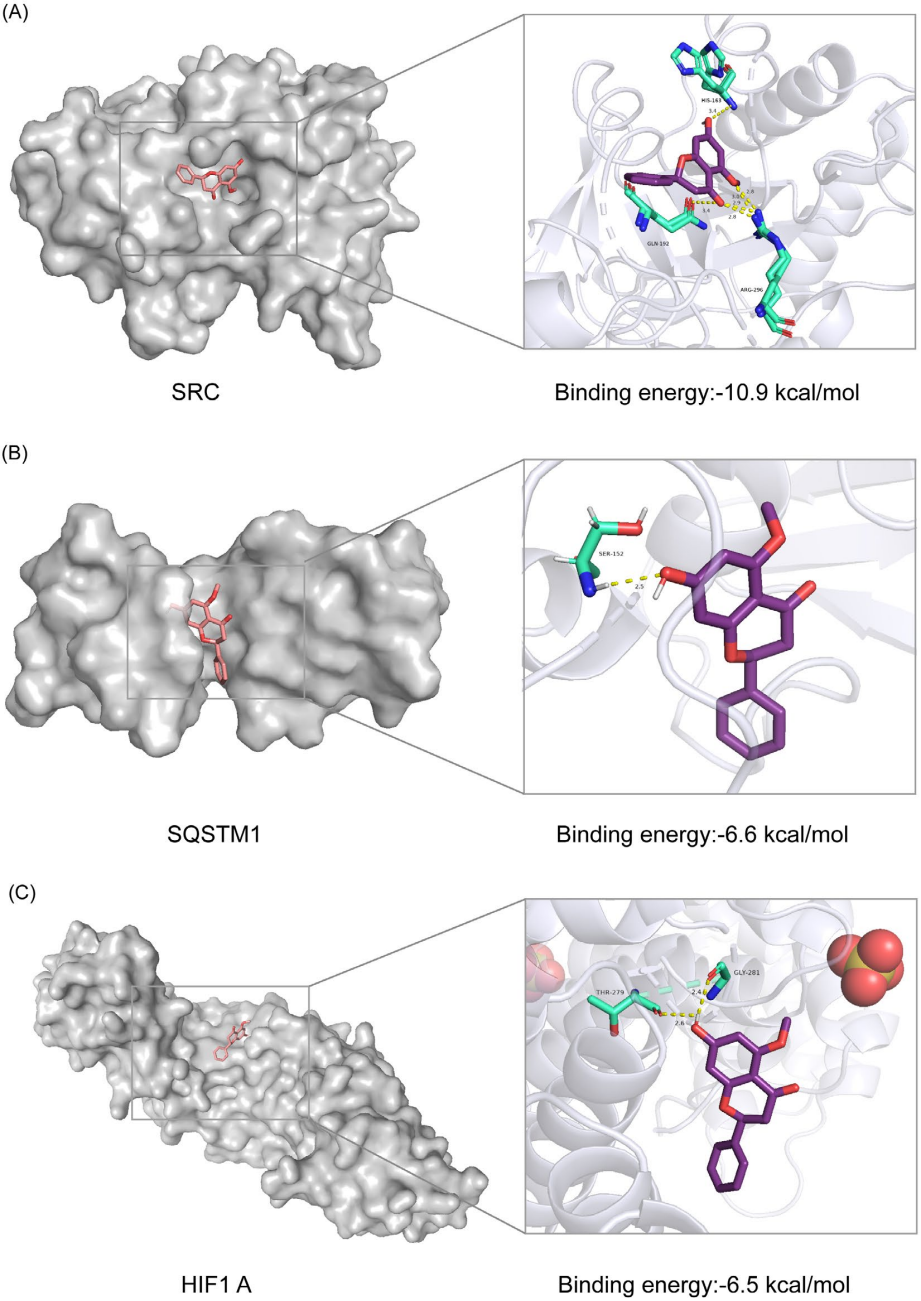
Docking patterns of key targets and Alpinetin. (A) SRC (1us0)‐Alpinetin; (B) SQSTM1 (5yp7)‐Alpinetin; (C) HIF1A (8he3)‐Alpinetin.

Three key targets—HIF1A, SQSTM1, and SRC—were identified based on the PPI interaction network and enrichment analysis, and the affinity of Alpinetin to them was verified through molecular docking. The necessary files were obtained from the PDB database, and all receptor files were processed to remove organic matter and water molecules, followed by the addition of hydrogen atoms and charge distributions. PyMOL was used for pretreatment, and molecular docking was performed using AutoDock Vina to obtain the docking results. A binding energy threshold of ≤ −5 kcal/mol (or pKd/pKi ≥ 5.52) is widely accepted as indicative of strong ligand‐protein affinity in molecular docking studies [41]. Consistent with this criterion, molecular docking analysis revealed that the binding energies for HIF1A (PDB ID: 8he3), SRC (PDB ID: 1us0), and SQSTM1 (PDB ID: 5yp7) were all ≤ −5 kcal/mol, demonstrating strong affinities between the ligand and these key target proteins. The results were visualized using PyMOL (Figure 5). This finding further confirms that Alpinetin acts on PD‐related core targets with high affinity, suggesting its potential to treat or improve PD through these interactions.

### 3.6 Alpinetin Significantly Improved Motor Deficits in MPTP‐Induced Mice

Network pharmacology and molecular docking analyses suggest that Alpinetin may exert therapeutic effects on PD through pathways involving dopaminergic synapses, mitophagy, and other molecular targets. To substantiate these predictions and elucidate the specific mechanisms, experimental validation was subsequently performed.

To establish a PD model, mice were administered MPTP, a neurotoxin that readily crosses the blood–brain barrier and is converted to its active metabolite, MPP^+^. MPP^+^ is selectively taken up by dopaminergic neurons via the dopamine transporter, leading to mitochondrial dysfunction, oxidative stress, and eventual neuronal death within the substantia nigra pars compacta and striatum [38]. This process recapitulates key features of PD, including significant motor deficits, profound striatal dopamine depletion, and neuropathological alterations consistent with the disease.

**FIGURE 6:**
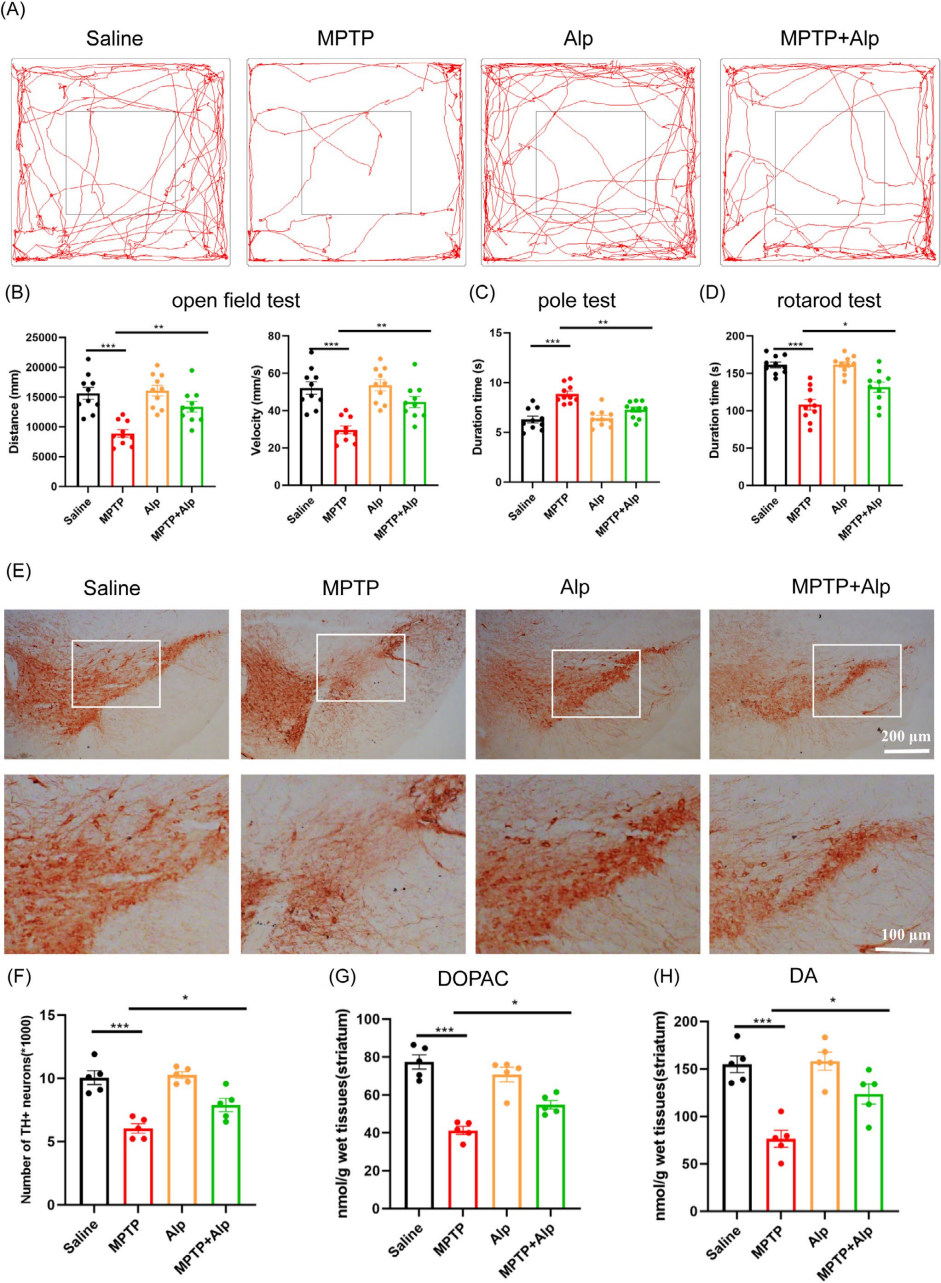
Protective effects of Alpinetin on MPTP‐induced PD animal models. (A) Tracking analysis of locomotor activity in open field test (*n* = 10). (B) Quantitative analysis of open field test: distance and velocity (*n* = 10). (C) Pole test (*n* = 10). (D) Rotarod test (*n* = 10). (E) TH Immunohistochemistry of dopaminergic neurons in the SN (scale bar = 200 μm/100 μm, *n* = 5). (F) Quantification of TH+ neurons in the SN. (G) Dopac levels in Striatum tissue (*n* = 5). (H) Dopamine levels in Striatum tissue. SN, Substantia Nigra (*n* = 5). *p < 0.05, **p < 0.01, ***p < 0.001 compared with the corresponding group, as determined by the one‐way ANOVA.

In PD, the destruction of dopamine neurons will lead to a series of motor symptoms such as bradykinesia and muscle rigidity [42]. Consequently, to evaluate the therapeutic effect of Alpinetin on PD, a series of behavioral tests were conducted in mice (Figure 6A–D). The results showed that MPTP‐injected mice exhibited a significant reduction in total movement distance and movement speed in the open field compared to control (saline‐treated) mice (Figure 6A,B). Additionally, MPTP‐induced mice spent less time on the rotarod (Figure 6D), experienced more frequent falls, and showed significantly prolonged motor duration in the pole test (Figure 6C). Notably, Alpinetin demonstrated a protective effect on motor function in MPTP‐induced mice. Mice treated with Alpinetin exhibited faster movement speeds compared to MPTP‐induced mice (Figure 6A,B). Furthermore, Alpinetin‐treated mice showed significant improvements in balance and pole‐climbing speed (Figure 6D).

To verify that the observed effects were due to the neuroprotective action of Alpinetin, rather than a direct enhancement of motor function, we repeated the same experiment in normal mice treated with Alpinetin. No improvement in locomotion was observed in these control subjects (Figure 6A–D). Therefore, we conclude that Alpinetin specifically enhances motor function within the context of the MPTP model.

### 3.7 Alpinetin Improved TH Neurons and Dopamine Loss in MPTP‐Induced Mice

Following the observation that Alpinetin significantly improved motor deficits in MPTP‐induced PD mice, we further explored its potential effects on underlying pathology. Specifically, we examined TH neurons and DA levels, which are essential biomarkers of dopaminergic function. TH is a key enzyme in dopamine synthesis and serves as a reliable marker for dopaminergic neuron health. DA, critical for motor function, is markedly reduced in PD, leading to the characteristic motor impairments of the disorder to elucidate the mechanisms underlying the effects of Alpinetin, we assessed changes in TH neuron integrity and DA levels.

Using TH immunohistochemical staining, we compared the number of TH‐positive neurons across the four groups of mice. Analysis of Figure 6E,F showed that both the control and Alpinetin‐treated groups exhibited a dense population of TH‐positive neurons, indicating normal neuronal distribution. In contrast, MPTP‐induced mice demonstrated a significant reduction in neuron count, reflecting the neurotoxic effects of MPTP. Although the combined treatment of MPTP and Alpinetin did not fully restore normal neuronal levels, the treatment group showed a significantly higher neuron count compared to the MPTP‐only group, suggesting that Alpinetin partially mitigated the neural damage caused by MPTP. Furthermore, HPLC analysis revealed that Alpinetin‐treated MPTP mice showed marked improvement in dopamine levels and its metabolite DOPAC (Figure 6G,H).

Overall, these findings suggest that Alpinetin has potential utility in preventing and treating MPTP‐induced PD, particularly by reducing neuronal loss and protecting the nervous system.

### 3.8 Alpinetin Enhances Mitophagy in MPTP‐Induced Mice

Based on initial predictions from network pharmacology enrichment analysis and molecular docking, we also propose that Alpinetin may exert therapeutic effects through the mitophagy pathway. Recent studies have implicated impaired mitophagy in the pathogenesis of neurodegenerative diseases, including PD. For instance, the accumulation of abnormal protein aggregates and disruption of mitochondrial homeostasis may exacerbate the degeneration of dopaminergic neurons through dysregulated autophagy pathways [43]. Previous studies confirmed its behavioral and neuroprotective effects in PD mice. To further elucidate the underlying mechanisms, we conducted experimental studies focusing on the mitophagy pathway.

**FIGURE 7:**
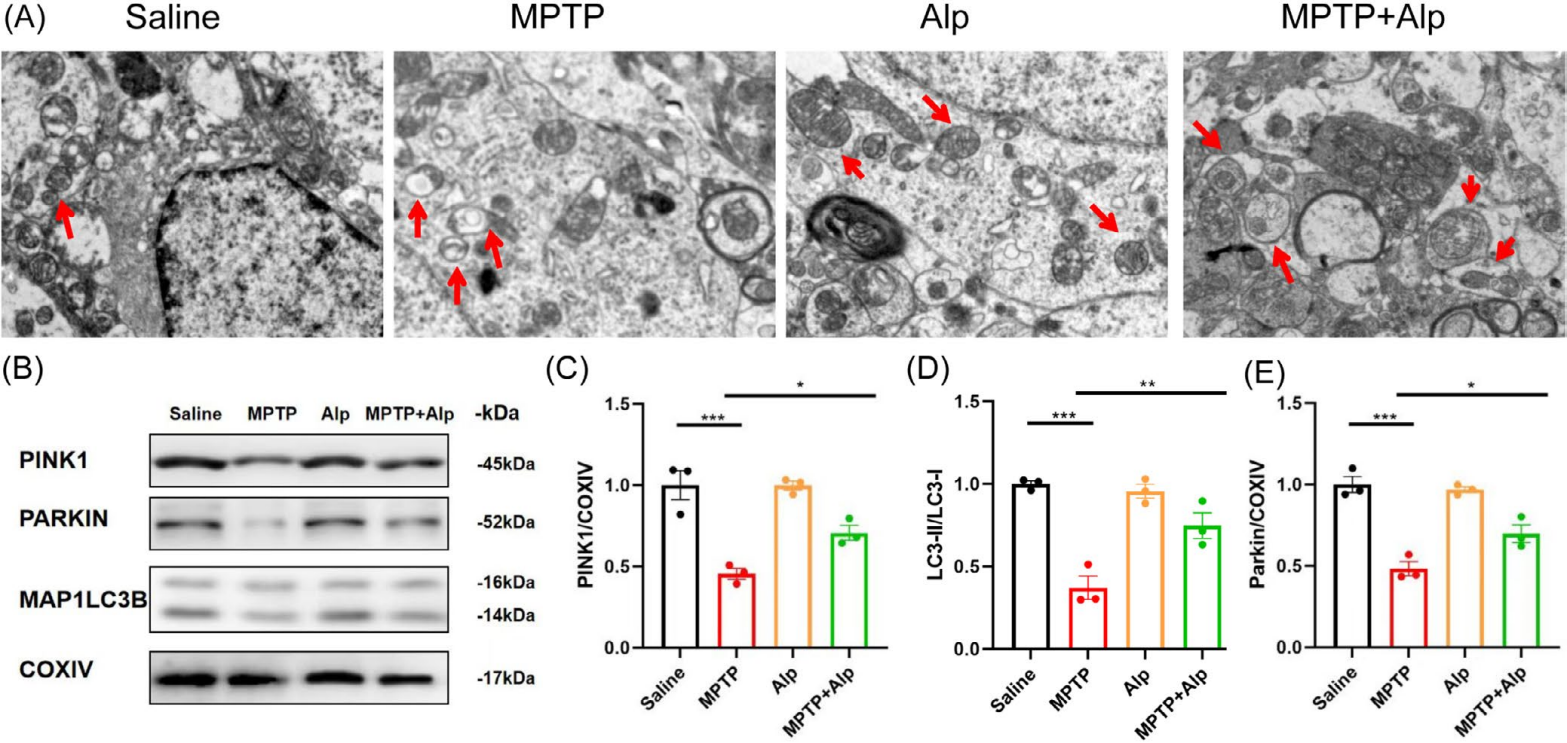
Alpinetin mitigates mitochondrial dysfunction and enhances mitophagy in the MPTP‐Induced PD Model. (A) Transmission electron microscopy of mitochondrial morphology in the SN. (B) WB analysis of PINK1, Parkin, and MAP1LC3B expression 3 independent replicate experiments were performed. (C) Quantification of PINK1. (D) Ratio of LC3‐II to LC3‐I contents. (E) Quantification of Parkin. PD, Parkinson's disease. SN, Substantia Nigra; WB, western blot. *p < 0.05, **p < 0.01, ***p < 0.001 compared with the corresponding group, as determined by the one‐way ANOVA.

Transmission electron microscopy was employed to examine mitophagy in midbrain tissue sections. In the control group, mitochondria appeared structurally intact, with well‐preserved configurations and clearly defined internal features, showing no damage. Conversely, midbrain mitochondria from MPTP‐induced mice displayed marked structural damage, including internal disintegration and irregular membranes, typical of MPTP‐induced mitochondrial toxicity. However, in the MPTP and Alpinetin co‐treatment group, mitochondrial structures were notably preserved, with increased signs of mitochondrial autophagy (Figure 7A), despite some residual damage. This suggests that Alpinetin exerts a protective effect against MPTP‐induced mitochondrial damage by enhancing mitochondrial autophagy, aiding in the clearance of damaged mitochondria to support cellular health.

To further investigate the regulatory role of Alpinetin in mitophagy, we assessed the expression levels of Parkin, PINK1, and LC3 in mouse brain tissues via Western blotting (Figure 7B–E). MPTP treatment markedly suppressed the PINK1‐Parkin pathway: Under physiological conditions, mitochondrial damage triggers PINK1 accumulation on the outer mitochondrial membrane, activating Parkin to ubiquitinate mitochondrial protein. Alpinetin restored the expression of both PINK1 and Parkin, indicating its ability to enhance the labeling and recognition of damaged mitochondria. Concurrently, Alpinetin significantly reversed the MPTP‐induced reduction in LC3 levels. LC3, a key component of autophagosome membranes, is associated with increased autophagosome formation when upregulated. This process not only facilitates the removal of dysfunctional mitochondria but also mitigates reactive oxygen species (ROS) accumulation in neurons, thereby preserving cellular energy homeostasis.

Overall, these results indicate that MPTP‐induced mitochondrial structural damage in mouse brain tissue may be mitigated by the protective mechanism of Alpinetin. Combined with the network pharmacology analysis, it can be speculated that Alpinetin may exert a protective effect on PD pathology in MPTP‐induced mice by enhancing mitophagy.

## 4 Discussion

PD is an incurable neurodegenerative disorder with a steadily increasing global incidence. Its complex pathogenesis has limited the success of many targeted therapies. In this study, a network pharmacology‐based approach was used to identify potential therapeutic targets of Alpinetin in PD. PPI network analysis was performed to explore the relationships among the identified targets. Core targets were then subjected to GO enrichment analysis, with results categorized into BP, CC, and MF. KEGG pathway analysis identified two key pathways: mitophagy‐related signaling and dopaminergic synaptic transmission. These findings suggest that Alpinetin may exert anti‐PD effects by modulating these pathways.

GO enrichment analysis identified BP, CC, and MF significantly associated with the pathogenesis of PD. Within the biological processes category, dysregulation of autophagy was found to be a central pathogenic mechanism underlying neurodegenerative disorders [44]. Deficiencies in the *PINK1/PARK2* pathway impair mitophagy, leading to the accumulation of aberrant proteins and damaged organelles, which ultimately contribute to Lewy body formation [45]. Mitochondrial dysfunction and oxidative stress are key pathological mechanisms in PD [46]. A dysregulated response to decreased oxygen levels, driven by mitochondrial respiratory chain impairment and ROS overproduction, exacerbates neuronal oxidative damage [47]. Aberrant lipid metabolism is also critically involved in neurodegenerative processes [48]. In PD, lipid peroxidation and compromised cellular membrane integrity may accelerate neuronal degeneration. For example, pathological aggregation of α‐synuclein disrupts lipid bilayer stability [49], while dysregulation of sphingolipid metabolism has been linked to neuroinflammation and autophagic dysfunction [50, 51]. From the cellular component perspective, damage to neuronal cell bodies is directly linked to the degenerative loss of dopaminergic neurons, where dysregulation of intracellular protein synthesis and transport—exemplified by *α*‐synuclein aggregation—results in the collapse of neuronal homeostasis [52]. Concurrently, structural degeneration of dendrites disrupts neuroinflammatory regulation, with dendritic cells (DCs) migrating to lymph nodes during neuroinflammatory processes, thus potentiating immune activation [53]. Regarding molecular function, the primary involvements include protein kinase binding, transcription factor binding, and nuclear receptor binding. Mutations in kinases such as leucine‐rich repeat kinase 2 (LRRK2) lead to selective degeneration of dopaminergic neurons, impairing neuronal survival and function, and directly precipitating familial PD [54]. Additionally, genes such as Parkin regulate transcription factors like nuclear factor erythroid 2‐related factor 2 (Nrf2) to sustain antioxidant defense and mitochondrial quality control. Loss of their binding capacity compromises neuronal defense against oxidative stress, thereby accelerating PD progression [55, 56]. The ligand‐binding activity of nuclear receptors regulates neuroinflammatory processes and metabolic homeostasis in PD. For example, activation of peroxisome proliferator‐activated receptor gamma (PPARγ) suppresses microglial hyperactivation, thereby reducing the release of pro‐inflammatory cytokines such as tumor necrosis factor‐alpha (TNF‐*α*) and interleukin‐1 beta (IL‐1β), which mitigates neuroinflammatory damage to dopaminergic neurons in PD. Furthermore, PPARγ enhances mitochondrial function and reduces oxidative stress, thereby providing neuroprotection. Experimental studies show that PPARγ agonists (e.g., pioglitazone) inhibit inflammatory responses by downregulating NLRP3 inflammasomes, suppressing nuclear factor kappa‐B (NF‐κB) signaling, and decreasing TNF‐*α* and cyclooxygenase‐2 (COX‐2) expression [57, 58]. The comprehensive GO terms reveal the molecular complexity of PD, where multilevel defects in signal transduction and gene regulation ultimately lead to systemic neurodegeneration. This integrated perspective provides a deeper understanding of PD pathogenesis and highlights multiple therapeutic pathways and targets for intervention.

Through KEGG enrichment analysis, HIF1A, SRC and SQSTM1 were identified as the key targets of Alpinetin on the mitophagy pathway. Previous studies have shown that Hypoxia‐Inducible Factor‐1 (HIF1) is a transcription factor stabilized and activated under hypoxic conditions, inducing the expression of various genes associated with cellular adaptive responses [59]. HIF1's role in regulating mitophagy primarily involves the activation of metabolic and autophagy‐related genes. Under hypoxic conditions, HIF1 upregulates mitophagy‐related genes such as BNIP3 and NIX, promoting the initiation of mitophagy [60]. BNIP3 facilitates the degradation of damaged mitochondria by tagging them for entry into autophagosomes [61]. By promoting mitophagy, HIF1 helps reduce the accumulation of damaged mitochondria, lowers ROS levels, and protects cells from oxidative stress. Furthermore, HIF1 accumulation can rescue DJ‐1‐deficient neurons from MPP + ‐induced toxicity, thereby mitigating oxidative stress and ROS damage in neuronal cells [62]. Src is a non‐receptor tyrosine kinase involved in various signaling pathways, particularly in the regulation of cell proliferation, survival, and apoptosis [63]. Studies have shown that inhibition of Src prevents the loss of TH‐positive neurons in the SNPC of MPTP‐induced mice and increases both mRNA and protein levels of TH, indicating a potent neuroprotective effect on dopaminergic neurons. Additionally, MPTP‐induced mice administered Src inhibitors exhibited significantly improved performance in behavioral tests [64], further supporting the neuroprotective potential of Src inhibition. In addition, SQSTM1 encodes the P62 protein, the first identified selective autophagy receptor [65, 66]. During mitophagy, p62 primarily functions by recognizing ubiquitinated signals generated through the PINK1‐Parkin pathway [67]. PINK1, a mitochondrial kinase containing an N‐terminal mitochondrial targeting sequence (MTS), localizes to the surface of damaged mitochondria, where its kinase activity is activated [68]. Parkin, a 465‐amino acid E3 ubiquitin ligase encoded by *PARK2*, operates downstream of PINK1. The accumulation of PINK1 on dysfunctional mitochondria facilitates the recruitment and activation of Parkin to depolarized mitochondrial surfaces. Activated Parkin subsequently catalyzes the ubiquitination of mitochondrial proteins [45, 69]. P62 mediates this process by directly binding to Parkin‐induced ubiquitinated mitochondrial substrates, followed by interaction with LC3 proteins on autophagosome membranes. This sequential binding event targets ubiquitinated mitochondria to autophagosomes for degradation, thereby completing the mitophagy process [67]. Studies have shown that mitophagy is dependent on the PINK1‐Parkin pathway, with mutations in PINK1 and Parkin representing the earliest genetic events linked to autosomal recessive early‐onset PD [70]. Additionally, p62 knockout does not impair Parkin recruitment to mitochondria but significantly delays the clearance of damaged mitochondria, elevates mitochondrial superoxide levels, and compromises mitochondrial respiratory function [71], thereby inducing PD‐related phenotypes. Molecular docking studies revealed that Alpinetin has a high binding affinity for HIF1A, SRC, and SQSTM1. Animal experiments further demonstrated that Alpinetin upregulates the expression of Parkin, PINK1, and LC3 proteins—critical mediators of mitophagy—in the brains of PD mice. These findings collectively suggest that the therapeutic effects of Alpinetin in PD mice are linked to the restoration of mitophagy function.

Mitophagy is a selective autophagic mechanism that specifically eliminates damaged or dysfunctional mitochondria, crucial for maintaining cellular homeostasis and preventing cellular aging and disease progression [43, 72]. Dysregulation of this process impairs various physiological functions and energy metabolism [73], particularly compromising the functionality of dopaminergic synapses [16]. Dopaminergic synapses, which use dopamine as the primary neurotransmitter in the central nervous system (CNS), are primarily located in the SNPC and ventral tegmental area. These neural projections extend to the striatum, prefrontal cortex, and other regions, playing a critical role in regulating motor control, reward mechanisms, cognition, and emotional modulation. The structural organization of dopaminergic synapses is centered on presynaptic terminals, which house key mechanisms governing dopamine synthesis, storage, and release [74, 75]. After dopamine release, reuptake is primarily mediated by the dopamine transporter (DAT), while any remaining unrecaptured dopamine undergoes oxidative deamination catalyzed by monoamine oxidase (MAO) in the cytosol or mitochondrial outer membranes [76], a process that prevents excessive dopamine accumulation under normal conditions. Critical synaptic functions, including synaptic vesicle endocytosis (SVE), dopamine reuptake, and neurotransmitter release, require substantial ATP supplies, mainly derived from mitochondrial oxidative phosphorylation [77, 78]. Normal mitophagy ensures synaptic energy homeostasis through the efficient clearance of dysfunctional mitochondria, thereby maintaining high‐fidelity ATP production essential for sustained synaptic activity. In addition, the maintenance of synaptic homeostasis directly feedbacks to protect mitochondrial integrity. Efficient synaptic operations, such as dopamine reuptake and synaptic vesicle endocytosis, prevent pathological excitotoxicity and a harmful accumulation of ROS. By averting this cascade of oxidative damage, a well‐functioning synapse alleviates the mitochondrial burden, reducing the source of damage it must clear. Thus, mitophagy and synaptic function engage in a virtuous cycle of mutual preservation, whose disruption may initiate a vicious cycle contributing to neurodegeneration [79]. However, in PD, cytosolic dopamine (DA) metabolism via MAO generates hydrogen peroxide byproducts that exacerbate ROS and produce neurotoxic quinones [76]. Additionally, reduced expression of *PINK1* and *PARK2* genes in PD compromises mitophagy clearance, leading to the progressive accumulation of dysfunctional mitochondria. This pathological cascade amplifies intracellular oxidative stress and energy deficits, accelerating dopaminergic neuronal degeneration [43, 80] and establishing a self‐amplifying vicious cycle. Current therapeutic strategies targeting PD have focused on enhancing mitophagy [81] and inhibiting MAO [82] to mitigate oxidative damage. In vivo experimental validation further demonstrated that Alpinetin ameliorates both motor and non‐motor behavioral deficits in PD mice, significantly attenuating DA neuronal degeneration.

In summary, our findings demonstrate that Alpinetin alleviates MPTP‐induced tyrosine hydroxylase‐positive (TH+) neuronal loss and DA damage, improving PD‐related symptoms by mitigating motor deficits, increasing DA neuron populations, and reducing mitophagy impairment. Mechanistically, Alpinetin targets HIF‐1, SRC, SQSTM1, MAO, and related pathways to counteract both mitophagy dysfunction and dopaminergic synaptic compromise. These results provide a solid theoretical foundation for the clinical translation of Alpinetin in PD management. Additionally, network pharmacology predictions combined with Western blot analyses reveal the multi‐target and multi‐pathway characteristics of Alpinetin. We hypothesize that other signaling pathways may contribute to its neuroprotective effects, although these mechanisms require further experimental validation.

It should be noted that the chronic MPTP model used in our study may not fully replicate PD progression. And while network pharmacology and molecular docking suggest multi‐target involvement, the precise regulatory mechanisms and binding specificities of Alpinetin require further validation using genetic knockdown approaches and more animal experiments. Meanwhile, future work should also explore its potential interactions with other PD‐related pathways, such as neuroinflammation or ferroptosis, to fully elucidate its neuroprotective profile.

## 5 Conclusion

In summary, this study elucidates the therapeutic potential of Alpinetin, a natural flavonoid, for treating PD through integrated bioinformatics, network pharmacology, and experimental validation approaches. Our findings identify Alpinetin as a multi‐target agent acting via 21 core targets, as revealed by PPI network analysis. GO and KEGG enrichment analyses indicate that these targets are predominantly associated with dopaminergic synapses and mitophagy pathways. Molecular docking studies further validate Alpinetin's stable binding affinity to three key proteins within these pathways. Experimental validation using an MPTP‐induced PD mouse model demonstrated that Alpinetin significantly improved motor function, reduced the loss of TH‐positive neurons, preserved dopamine levels, and alleviated mitophagy impairment. This study provides a foundational framework for understanding Alpinetin's multi‐target and multi‐pathway pharmacological mechanisms, suggesting its neuroprotective potential in PD. To further translate these findings, future studies will focus on validating the efficacy of Alpinetin in PD patient‐derived cell models and exploring its potential for combined therapy with other mitochondrial protectants.

## Author Contributions

**Zilu Shen:** conceptualization, methodology, and writing – original draft preparation. **Xuesong Shan:** supervision, methodology, software, and data curation. **Shenglan Zhang:** software, data curation, and writing – original draft preparation. **Dan Huang:** investigation and supervision. **Haijun Hu and Yonglin Liang:** supervision and software. **Hong Zhu:** software and investigation. **Yayu Chen and Lieliang Zhang:** funding acquisition, project administration, and writing – review and editing.

## Funding

This work was supported by the National Natural Science Foundation of China (82360227, 82560229), the Natural Science Foundation of Jiangxi Province (20232BAB206060, 20252BAC240543, 20242BAB20470), and the Jiangxi Provincial Outstanding Young Talent Fund (20252BAC220059).

## Ethics Statement

The current study does not involve human participants, human tissue or human data. All animal procedures were conducted in accordance with the Chinese Guidelines of Animal Care and Welfare, as well as the guidelines of the Institutional Animal Care and Use Committee of Nanchang University (Approval number: RYE2024032901).

## Consent

All authors have read and approved the final manuscript.

## Conflicts of Interest

The authors declare no conflicts of interest.

## Data Availability

All data analyzed and presented in this study is available from the corresponding author on reasonable request.

## References

[1] C. M. Tanner and J. L. Ostrem , “Parkinson's Disease,” New England Journal of Medicine 391 (2024): 442–452, 10.1056/NEJMra2401857. 39083773

[2] B. R. Bloem , M. S. Okun , and C. Klein , “Parkinson's Disease,” Lancet 397 (2021): 2284–2303, 10.1016/S0140-6736(21)00218-X. 33848468

[3] M. J. Armstrong and M. S. Okun , “Diagnosis and Treatment of Parkinson Disease: A Review,” JAMA 323 (2020): 548–560, 10.1001/jama.2019.22360. 32044947

[4] A. H. V. Schapira , M. Emre , P. Jenner , and W. Poewe , “Levodopa in the Treatment of Parkinson's Disease,” European Journal of Neurology 16 (2009): 982–989, 10.1111/j.1468-1331.2009.02697.x. 19538218

[5] D. Lin , Y. Zeng , D. Tang , and Y. Cai , “Study on the Mechanism of Liuwei Dihuang Pills in Treating Parkinson's Disease Based on Network Pharmacology,” BioMed Research International 2021 (2021): 4490081, 10.1155/2021/4490081. 34746302 PMC8568527

[6] Y. Wu , H. Liu , Y. Wang , et al., “DiHuangYin Decoction Protects Dopaminergic Neurons in a Parkinson's Disease Model by Alleviating Peripheral Inflammation,” Phytomedicine 105 (2022): 154357, 10.1016/j.phymed.2022.154357. 35933898

[7] J. Moreira , M. Machado , M. Dias‐Teixeira , R. Ferraz , C. Delerue‐Matos , and C. Grosso , “The Neuroprotective Effect of Traditional Chinese Medicinal Plants—A Critical Review,” Acta Pharmaceutica Sinica B 13 (2023): 3208–3237, 10.1016/j.apsb.2023.06.009. 37655317 PMC10465969

[8] S. Gul , M. F. Maqbool , D. Zheng , Y. Li , M. Khan , and T. Ma , “Alpinetin: A Dietary Flavonoid With Diverse Anticancer Effects,” Applied Biochemistry and Biotechnology 194 (2022): 4220–4243, 10.1007/s12010-022-03960-2. 35567708

[9] G. Zhao , Y. Tong , F. Luan , et al., “Alpinetin: A Review of Its Pharmacology and Pharmacokinetics,” Frontiers in Pharmacology 13 (2022): 814370, 10.3389/fphar.2022.814370. 35185569 PMC8854656

[10] K. Hu , Y. Yang , Q. Tu , Y. Luo , and R. Ma , “Alpinetin Inhibits LPS‐Induced Inflammatory Mediator Response by Activating PPAR‐γ in THP‐1‐Derived Macrophages,” European Journal of Pharmacology 721 (2013): 96–102, 10.1016/j.ejphar.2013.09.049. 24104193

[11] S. Xiao , Y. Zhang , Z. Liu , et al., “Alpinetin Inhibits Neuroinflammation and Neuronal Apoptosis via Targeting the JAK2/STAT3 Signaling Pathway in Spinal Cord Injury,” CNS Neuroscience & Therapeutics 29 (2023): 1094–1108, 10.1111/cns.14085. 36627822 PMC10018110

[12] M. G. Tansey and M. S. Goldberg , “Neuroinflammation in Parkinson's Disease: Its Role in Neuronal Death and Implications for Therapeutic Intervention,” Neurobiology of Disease 37 (2010): 510–518, 10.1016/j.nbd.2009.11.004. 19913097 PMC2823829

[13] Y. Chen , C. Yang , M. Zou , et al., “Inhibiting Mitochondrial Inflammation Through Drp1/ HK1 / NLRP3 Pathway: A Mechanism of Alpinetin Attenuated Aging‐Associated Cognitive Impairment,” Phytotherapy Research 37 (2023): 2454–2471, 10.1002/ptr.7767. 36772986

[14] M. Nguyen , Y. C. Wong , D. Ysselstein , A. Severino , and D. Krainc , “Synaptic, Mitochondrial, and Lysosomal Dysfunction in Parkinson's Disease,” Trends in Neurosciences 42 (2019): 140–149, 10.1016/j.tins.2018.11.001. 30509690 PMC6452863

[15] H. Ye , L. A. Robak , M. Yu , M. Cykowski , and J. M. Shulman , “Genetics and Pathogenesis of Parkinson's Syndrome,” Annual Review of Pathology: Mechanisms of Disease 18 (2023): 95–121, 10.1146/annurev-pathmechdis-031521-034145. PMC10290758 36100231

[16] G. Lou , K. Palikaras , S. Lautrup , M. Scheibye‐Knudsen , N. Tavernarakis , and E. F. Fang , “Mitophagy and Neuroprotection,” Trends in Molecular Medicine 26 (2020): 8–20, 10.1016/j.molmed.2019.07.002. 31375365

[17] J. Qiu , Y. Chen , J. Zhuo , et al., “Urolithin A Promotes Mitophagy and Suppresses NLRP3 Inflammasome Activation in Lipopolysaccharide‐Induced BV2 Microglial Cells and MPTP‐Induced Parkinson's Disease Model,” Neuropharmacology 207 (2022): 108963, 10.1016/j.neuropharm.2022.108963. 35065082

[18] M. Onishi , K. Yamano , M. Sato , N. Matsuda , and K. Okamoto , “Molecular Mechanisms and Physiological Functions of Mitophagy,” EMBO Journal 40 (2021): e104705, 10.15252/embj.2020104705. 33438778 PMC7849173

[19] Y. Lu , Z. Li , S. Zhang , T. Zhang , Y. Liu , and L. Zhang , “Cellular Mitophagy: Mechanism, Roles in Diseases and Small Molecule Pharmacological Regulation,” Theranostics 13 (2023): 736–766, 10.7150/thno.79876. 36632220 PMC9830443

[20] A. B. Malpartida , M. Williamson , D. P. Narendra , R. Wade‐Martins , and B. J. Ryan , “Mitochondrial Dysfunction and Mitophagy in Parkinson's Disease: From Mechanism to Therapy,” Trends in Biochemical Sciences 46 (2021): 329–343, 10.1016/j.tibs.2020.11.007. 33323315

[21] A. L. Hopkins , “Network Pharmacology,” Nature Biotechnology 25 (2007): 1110–1111, 10.1038/nbt1007-1110. 17921993

[22] A. Friboulet and D. Thomas , “Systems Biology—An Interdisciplinary Approach,” Biosensors and Bioelectronics 20 (2005): 2404–2407, 10.1016/j.bios.2004.11.014. 15854815

[23] A.‐L. Barabási , N. Gulbahce , and J. Loscalzo , “Network Medicine: A Network‐Based Approach to Human Disease,” Nature Reviews Genetics 12 (2011): 56–68, 10.1038/nrg2918. PMC3140052 21164525

[24] J. M. Paggi , A. Pandit , and R. O. Dror , “The Art and Science of Molecular Docking,” Annual Review of Biochemistry 93 (2024): 389–410, 10.1146/annurev-biochem-030222-120000. PMC13198409 38594926

[25] “Home – OMIM,” 2023, https://www.omim.org/.

[26] “GeneCards ‐ Human Genes | Gene Database | Gene Search,” 2023, https://www.genecards.org/.

[27] “The Most Extensive & Reliable Gene‐Disease Database | DISGENET,” 2023, https://disgenet.com/.

[28] “TTD: Therapeutic Target Database,” 2023, https://db.idrblab.net/ttd/.

[29] “PharmMapper,” 2023, https://www.lilab‐ecust.cn/pharmmapper/.

[30] “SwissTargetPrediction,” 2023, http://www.swisstargetprediction.ch/.

[31] “ChEMBL – ChEMBL,” 2023, https://www.ebi.ac.uk/chembl/.

[32] “SEA,” 2023, http://sea.edbc.org/.

[33] X. Kong , C. Liu , Z. Zhang , et al., “BATMAN‐TCM 2.0: An Enhanced Integrative Database for Known and Predicted Interactions Between Traditional Chinese Medicine Ingredients and Target Proteins,” Nucleic Acids Research 52 (2024): D1110–D1120, 10.1093/nar/gkad926. 37904598 PMC10767940

[34] “The Comparative Toxicogenomics Database | CTD,” 2023, https://ctdbase.org/.

[35] “The Microbioinformatics Online Platform,” 2023, https://www.bioinformatics.com.cn/.

[36] “Metascape,” 2023, https://metascape.org/gp/index.html#/main/step1.

[37] A. Isenbrandt , M. Morissette , M. Bourque , et al., “Effect of Sex and Gonadectomy on Brain MPTP Toxicity and Response to Dutasteride Treatment in Mice,” Neuropharmacology 201 (2021): 108784, 10.1016/j.neuropharm.2021.108784. 34555366

[38] M. Mustapha and C. N. Mat Taib , “MPTP‐Induced Mouse Model of Parkinson's Disease: A Promising Direction of Therapeutic Strategies,” Bosnian Journal of Basic Medical Sciences 21 (2020): 422–433, 10.17305/bjbms.2020.5181. PMC8292858 33357211

[39] S. Kim , S.‐H. Kwon , T.‐I. Kam , et al., “Transneuronal Propagation of Pathologic α‐Synuclein From the Gut to the Brain Models Parkinson's Disease,” Neuron 103 (2019): 627–641.e7, 10.1016/j.neuron.2019.05.035. 31255487 PMC6706297

[40] K. C. Luk , V. Kehm , J. Carroll , et al., “Pathological α‐Synuclein Transmission Initiates Parkinson‐Like Neurodegeneration in Nontransgenic Mice,” Science 338 (2012): 949–953, 10.1126/science.1227157. 23161999 PMC3552321

[41] K.‐Y. Hsin , S. Ghosh , and H. Kitano , “Combining Machine Learning Systems and Multiple Docking Simulation Packages to Improve Docking Prediction Reliability for Network Pharmacology,” PLoS One 8 (2013): e83922, 10.1371/journal.pone.0083922. 24391846 PMC3877102

[42] L. V. Kalia and A. E. Lang , “Parkinson's Disease,” Lancet 386 (2015): 896–912, 10.1016/S0140-6736(14)61393-3. 25904081

[43] A. Picca , J. Faitg , J. Auwerx , L. Ferrucci , and D. D'Amico , “Mitophagy in Human Health, Ageing and Disease,” Nature Metabolism 5 (2023): 2047–2061, 10.1038/s42255-023-00930-8. PMC12159423 38036770

[44] T. Hara , K. Nakamura , M. Matsui , et al., “Suppression of Basal Autophagy in Neural Cells Causes Neurodegenerative Disease in Mice,” Nature 441 (2006): 885–889, 10.1038/nature04724. 16625204

[45] A. M. Pickrell and R. J. Youle , “The Roles of PINK1, Parkin, and Mitochondrial Fidelity in Parkinson's Disease,” Neuron 85 (2015): 257–273, 10.1016/j.neuron.2014.12.007. 25611507 PMC4764997

[46] S. R. Subramaniam and M.‐F. Chesselet , “Mitochondrial Dysfunction and Oxidative Stress in Parkinson's Disease,” Progress in Neurobiology 106 (2013): 17–32, 10.1016/j.pneurobio.2013.04.004. 23643800 PMC3742021

[47] P. Lee , N. S. Chandel , and M. C. Simon , “Cellular Adaptation to Hypoxia Through Hypoxia Inducible Factors and Beyond,” Nature Reviews Molecular Cell Biology 21 (2020): 268–283, 10.1038/s41580-020-0227-y. 32144406 PMC7222024

[48] L. G. Yang , Z. M. March , R. A. Stephenson , and P. S. Narayan , “Apolipoprotein E in Lipid Metabolism and Neurodegenerative Disease,” Trends in Endocrinology and Metabolism 34 (2023): 430–445, 10.1016/j.tem.2023.05.002. 37357100 PMC10365028

[49] B. A. Killinger , R. Melki , P. Brundin , and J. H. Kordower , “Endogenous Alpha‐Synuclein Monomers, Oligomers and Resulting Pathology: Let's Talk About the Lipids in the Room,” Npj Parkinson's Disease 5 (2019): 23, 10.1038/s41531-019-0095-3. PMC6851126 31728405

[50] Y. A. Hannun and L. M. Obeid , “Sphingolipids and Their Metabolism in Physiology and Disease,” Nature Reviews Molecular Cell Biology 19 (2018): 175–191, 10.1038/nrm.2017.107. 29165427 PMC5902181

[51] Y. A. Hannun and L. M. Obeid , “Author Correction: Sphingolipids and Their Metabolism in Physiology and Disease,” Nature Reviews Molecular Cell Biology 19 (2018): 673, 10.1038/s41580-018-0046-6. 30111875

[52] H. A. Lashuel , C. R. Overk , A. Oueslati , and E. Masliah , “The Many Faces of α‐Synuclein: From Structure and Toxicity to Therapeutic Target,” Nature Reviews Neuroscience 14 (2013): 38–48, 10.1038/nrn3406. 23254192 PMC4295774

[53] T. Worbs , S. I. Hammerschmidt , and R. Förster , “Dendritic Cell Migration in Health and Disease,” Nature Reviews Immunology 17 (2017): 30–48, 10.1038/nri.2016.116. 27890914

[54] Y. Xiong and J. Yu , “LRRK2 in Parkinson's Disease: Upstream Regulation and Therapeutic Targeting,” Trends in Molecular Medicine 30 (2024): 982–996, 10.1016/j.molmed.2024.07.003. 39153957 PMC11466701

[55] A. T. Dinkova‐Kostova , R. V. Kostov , and A. G. Kazantsev , “The Role of Nrf2 Signaling in Counteracting Neurodegenerative Diseases,” FEBS Journal 285 (2018): 3576–3590, 10.1111/febs.14379. 29323772 PMC6221096

[56] S. Murakami , Y. Kusano , K. Okazaki , T. Akaike , and H. Motohashi , “NRF2 Signalling in Cytoprotection and Metabolism,” British Journal of Pharmacology (2023): bph.16246, 10.1111/bph.16246. 37715470

[57] H. Usman , Z. Tan , M. Gul , et al., “Identification of Novel and Potential PPARγ Stimulators as Repurposed Drugs for MCAO Associated Brain Degeneration,” Toxicology and Applied Pharmacology 446 (2022): 116055, 10.1016/j.taap.2022.116055. 35550883

[58] C. Titus , M. T. Hoque , and R. Bendayan , “PPAR Agonists for the Treatment of Neuroinflammatory Diseases,” Trends in Pharmacological Sciences 45 (2024): 9–23, 10.1016/j.tips.2023.11.004. 38065777

[59] G. L. Wang , B. H. Jiang , E. A. Rue , and G. L. Semenza , “Hypoxia‐Inducible Factor 1 Is a Basic‐Helix‐Loop‐Helix‐PAS Heterodimer Regulated by Cellular O2 Tension,” Proceedings of the National Academy of Sciences 92 (1995): 5510–5514, 10.1073/pnas.92.12.5510. PMC41725 7539918

[60] G. Bellot , R. Garcia‐Medina , P. Gounon , et al., “Hypoxia‐Induced Autophagy Is Mediated Through Hypoxia‐Inducible Factor Induction of BNIP3 and BNIP3L via Their BH3 Domains,” Molecular and Cellular Biology 29 (2009): 2570–2581, 10.1128/MCB.00166-09. 19273585 PMC2682037

[61] M. O. Gok , O. M. Connor , X. Wang , et al., “The Outer Mitochondrial Membrane Protein TMEM11 Demarcates Spatially Restricted BNIP3/BNIP3L‐Mediated Mitophagy,” Journal of Cell Biology 222 (2023): e202204021, 10.1083/jcb.202204021. 36795401 PMC9960330

[62] M. Parsanejad , Y. Zhang , D. Qu , et al., “Regulation of the VHL/HIF‐1 Pathway by DJ‐1,” Journal of Neuroscience 34 (2014): 8043–8050, 10.1523/JNEUROSCI.1244-13.2014. 24899725 PMC6608259

[63] R. Ishizawar and S. J. Parsons , “C‐Src and Cooperating Partners in Human Cancer,” Cancer Cell 6 (2004): 209–214, 10.1016/j.ccr.2004.09.001. 15380511

[64] H. Yang , L. Wang , C. Zang , et al., “Src Inhibition Attenuates Neuroinflammation and Protects Dopaminergic Neurons in Parkinson's Disease Models,” Frontiers in Neuroscience 14 (2020): 45, 10.3389/fnins.2020.00045. 32132891 PMC7040487

[65] S. Geisler , K. M. Holmström , D. Skujat , et al., “PINK1/Parkin‐Mediated Mitophagy Is Dependent on VDAC1 and p62/SQSTM1,” Nature Cell Biology 12 (2010): 119–131, 10.1038/ncb2012. 20098416

[66] T. Yamada , D. Murata , Y. Adachi , et al., “Mitochondrial Stasis Reveals p62‐Mediated Ubiquitination in Parkin‐Independent Mitophagy and Mitigates Nonalcoholic Fatty Liver Disease,” Cell Metabolism 28 (2018): 588–604, 10.1016/j.cmet.2018.06.014. 30017357 PMC6170673

[67] J. Li , D. Yang , Z. Li , et al., “PINK1/Parkin‐Mediated Mitophagy in Neurodegenerative Diseases,” Ageing Research Reviews 84 (2023): 101817, 10.1016/j.arr.2022.101817. 36503124

[68] R. Han , Y. Liu , S. Li , X.‐J. Li , and W. Yang , “PINK1‐PRKN Mediated Mitophagy: Differences Between *In Vitro* and *In Vivo* Models,” Autophagy 19 (2023): 1396–1405, 10.1080/15548627.2022.2139080. 36282767 PMC10240983

[69] D. P. Narendra , S. M. Jin , A. Tanaka , et al., “PINK1 Is Selectively Stabilized on Impaired Mitochondria to Activate Parkin,” PLoS Biology 8 (2010): e1000298, 10.1371/journal.pbio.1000298. 20126261 PMC2811155

[70] E. M. Valente , P. M. Abou‐Sleiman , V. Caputo , et al., “Hereditary Early‐Onset Parkinson's Disease Caused by Mutations in *PINK1* ,” Science 304 (2004): 1158–1160, 10.1126/science.1096284. 15087508

[71] T. D. Nguyen , S. Shaid , O. Vakhrusheva , et al., “Loss of the Selective Autophagy Receptor p62 Impairs Murine Myeloid Leukemia Progression and Mitophagy,” Blood 133 (2019): 168–179, 10.1182/blood-2018-02-833475. 30498063

[72] S. Wang , H. Long , L. Hou , et al., “The Mitophagy Pathway and Its Implications in Human Diseases,” Signal Transduction and Targeted Therapy 8 (2023): 304, 10.1038/s41392-023-01503-7. 37582956 PMC10427715

[73] J. B. Spinelli and M. C. Haigis , “The Multifaceted Contributions of Mitochondria to Cellular Metabolism,” Nature Cell Biology 20 (2018): 745–754, 10.1038/s41556-018-0124-1. 29950572 PMC6541229

[74] D. Sulzer , S. J. Cragg , and M. E. Rice , “Striatal Dopamine Neurotransmission: Regulation of Release and Uptake,” Basal Ganglia 6 (2016): 123–148, 10.1016/j.baga.2016.02.001. 27141430 PMC4850498

[75] K. M. L. Cramb , D. Beccano‐Kelly , S. J. Cragg , and R. Wade‐Martins , “Impaired Dopamine Release in Parkinson's Disease,” Brain 146 (2023): 3117–3132, 10.1093/brain/awad064. 36864664 PMC10393405

[76] J. Meiser , D. Weindl , and K. Hiller , “Complexity of Dopamine Metabolism,” Cell Communication and Signaling 11 (2013): 34, 10.1186/1478-811X-11-34. 23683503 PMC3693914

[77] A. K. Reeve , J. P. Grady , E. M. Cosgrave , et al., “Mitochondrial Dysfunction Within the Synapses of Substantia Nigra Neurons in Parkinson's Disease,” Npj Parkinson's Disease 4 (2018): 9, 10.1038/s41531-018-0044-6. PMC5979968 29872690

[78] V. Rangaraju , N. Calloway , and T. A. Ryan , “Activity‐Driven Local ATP Synthesis Is Required for Synaptic Function,” Cell 156 (2014): 825–835, 10.1016/j.cell.2013.12.042. 24529383 PMC3955179

[79] M. J. Devine and J. T. Kittler , “Mitochondria at the Neuronal Presynapse in Health and Disease,” Nature Reviews Neuroscience 19 (2018): 63–80, 10.1038/nrn.2017.170. 29348666

[80] L. E. Newman and G. S. Shadel , “Pink1/Parkin Link Inflammation, Mitochondrial Stress, and Neurodegeneration,” Journal of Cell Biology 217 (2018): 3327–3329, 10.1083/jcb.201808118. 30154188 PMC6168260

[81] L.‐K. Wu , S. Agarwal , C.‐H. Kuo , et al., “Artemisia Leaf Extract Protects Against Neuron Toxicity by TRPML1 Activation and Promoting Autophagy/Mitophagy Clearance in Both In Vitro and In Vivo Models of MPP+/MPTP‐Induced Parkinson's Disease,” Phytomedicine 104 (2022): 154250, 10.1016/j.phymed.2022.154250. 35752074

[82] Y.‐Y. Tan , P. Jenner , and S.‐D. Chen , “Monoamine Oxidase‐B Inhibitors for the Treatment of Parkinson's Disease: Past, Present, and Future,” Journal of Parkinson's Disease 12 (2022): 477–493, 10.3233/JPD-212976. PMC8925102 34957948

